# Selection for Postponed Senescence in *Drosophila melanogaster* Reveals Distinct Metabolic Aging Trajectories Modifiable by the Angiotensin‐Converting Enzyme Inhibitor Lisinopril

**DOI:** 10.1111/acel.70375

**Published:** 2026-01-14

**Authors:** Denise Vecchié, Robert R. H. Anholt, Trudy F. C. Mackay, Maria De Luca

**Affiliations:** ^1^ Department of Nutrition Sciences University of Alabama at Birmingham Birmingham Alabama USA; ^2^ Institute for Human Genetics and Department of Genetics and Biochemistry Clemson University Greenwood South Carolina USA

**Keywords:** energy metabolism, longevity, metabolomics, postponed reproductive senescence, renin‐angiotensin system blockade

## Abstract

Aging is accompanied by profound changes in energy metabolism, yet the underlying drivers and modulators of these shifts remain incompletely understood. Here, we investigated how life‐history evolution shapes metabolic aging and pharmacological responsiveness by leveraging *Drosophila melanogaster* lines divergently selected for reproductive timing. We measured organismal oxygen consumption rate and performed untargeted metabolomics in young and old flies of both sexes from long‐lived “O” lines (selected for female late‐life reproduction) and unselected “B” control lines. Males and females from the O lines maintained stable metabolic rates and largely preserved metabolite profiles with age, whereas B line flies showed age‐related increases in oxygen consumption, citrate accumulation, and elevated levels of medium‐ and long‐chain fatty acids, hallmarks of mitochondrial inefficiency and impaired lipid oxidation. Aged B flies also displayed elevated S‐adenosylmethionine, reduced sarcosine, and diminished heme levels, indicating dysregulation of one‐carbon metabolism and impaired heme biosynthesis. Furthermore, Vitamin B6 metabolites, pyridoxamine, pyridoxal, and 4‐pyridoxate, increased with aging only in B line females. Motivated by evidence implicating the renin‐angiotensin system in metabolic aging, we treated flies with the angiotensin‐converting enzyme (ACE) inhibitor lisinopril. Lisinopril prevented the age‐related rise in metabolic rate in B line females, aligning their metabolic phenotype with that of O line flies. This suggests that ACE inhibition may buffer against age‐associated increases in metabolic rate and contribute to enhanced metabolic stability. Our results show that selection for delayed reproduction and increased lifespan modifies age‐related metabolic trajectories and modulates physiological responses to pharmacological intervention.

## Graphical abstract

Assessment of oxygen consumption rates in young, middle‐aged, and old flies from the *Drosophila* B lines and long‐lived O lines revealed a significant increase in metabolic rate with aging only in B flies. Lisinopril prevented the age‐related rise in metabolic rate in B female flies. Untargeted metabolomics revealed distinct age‐related metabolic signatures between the lines.

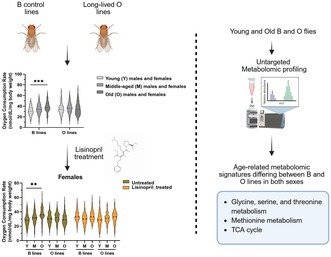

## 1 Introduction

Experimental evolution studies in *Drosophila melanogaster* have demonstrated that selecting for delayed reproduction in females extends lifespan. This provides empirical support for evolutionary theories of aging that posit a trade‐off between early‐life reproductive investment and late‐life somatic maintenance (Flatt and Partridge 2018). In the well‐characterized long‐lived “O” lines, originally developed by Rose (1984) and maintained under the same selection regime for over 30 years in the Mackay laboratory (Carnes et al. 2015), selection for reproduction at later ages in females over hundreds of generations has resulted in delayed female reproductive senescence and two‐fold increased lifespan in both sexes (Carnes et al. 2015). These lines, maintained in parallel with unselected “B” controls that reproduce on a standard 2‐week generation interval, provide a powerful model for dissecting the genetic architecture of aging, senescence, and longevity.

Genomic and transcriptomic comparisons between O and B lines have identified numerous candidate genes implicated in lifespan regulation of both females and males, including genes involved in stress response, immune function, mitochondrial activity, and metabolic regulation (Carnes et al. 2015). Notably, naturally occurring genetic variation in the promoter of *bellwether*, which encodes the α subunit of mitochondrial ATP synthase, modulates gene expression and lifespan in a sex‐specific manner (Garcia et al. 2017) and is genetically divergent in the long‐lived O lines relative to the B lines (Carnes et al. 2015). More recently, metabolomics studies have further shed light on the metabolic consequences of life‐history evolution. Hubert et al. (2025) reported that selection for early reproduction leads to accelerated aging and widespread metabolic remodeling, including alterations in mitochondrial function and lipid metabolism. In a related study, Phillips and collaborators (Phillips et al. 2022) combined experimental evolution with metabolomics to demonstrate that differences in longevity among *Drosophila* populations are associated with distinct metabolic signatures. Furthermore, Parkhitko et al. (2020) assessed metabolomic profiles in males of one B line (B3) and two O lines (O1 and O3) at 1 and 4 weeks of age, and observed significant age‐related changes in several metabolites involved in tryptophan metabolism, NADPH, and nucleotide metabolism. These findings highlight the value of integrating evolutionary and metabolic data to uncover conserved pathways that influence aging processes and longevity. However, while the above studies have revealed key genetic and biochemical differences, it remains unclear whether such longevity benefits arise from changes in basal energy metabolism or broader shifts in metabolic pathways that influence energy allocation and stress resilience. To address this, we investigated whether selection for delayed reproductive senescence in females alters the trajectory of metabolic aging in both sexes using the long‐lived O lines and their unselected B controls. Because symptoms of aging impairment, particularly in cardiac and locomotor function, begin to appear around 5 weeks of age in *D. melanogaster* (He and Jasper 2014), we first measured organismal oxygen consumption as a proxy for whole‐body metabolic rate across three life stages (1, 3, and 5 weeks of age). Subsequently, we performed untargeted metabolomics in 1‐ and 5‐week‐old flies to detect systemic differences in metabolite abundance associated with evolved longevity.

The renin‐angiotensin system (RAS), classically known for its role in regulating blood pressure and fluid balance, has recently garnered attention for its broader influence on metabolic homeostasis (Kanugula et al. 2023). Core components of the RAS are evolutionarily conserved across metazoans (Salzet et al. 2001), and genetic or pharmacological interventions targeting RAS have been shown to influence lifespan and healthspan in both invertebrate (Egan et al. 2024; Gabrawy et al. 2019; Gabrawy et al. 2022) and rodent (Benigni et al. 2009; de Cavanagh et al. 2024) models, highlighting a potential link between RAS suppression and metabolic resilience in aging. Previously, we revealed that the ACE inhibitor lisinopril, one of the most prescribed antihypertensive drugs (Olvera Lopez et al. 2024), modulates metabolic rate, glycogen content, and glycolytic throughput in *D. melanogaster* (Ederer et al. 2018; Vecchie et al. 2024). Using tissue‐specific knockdown of *Ance* (the primary *Drosophila* ortholog of the human *ACE* gene) in key metabolic tissues, we also revealed that lisinopril‐induced changes in whole‐body metabolic rate are *Ance*‐dependent, although the direction and magnitude of the effect vary across tissue, sex, and age (Vecchie et al. 2024). To determine whether genetic background shaped by selection for delayed reproduction modulates the physiological response to metabolic intervention, we assessed the effects of lisinopril treatment on organismal metabolic rate in 1‐, 3‐, and 5‐week‐old O and B flies.

Together, our integrated physiological, biochemical, and pharmacological analyses provide novel insights into how female reproductive timing and metabolic regulation interact to influence aging trajectories in both sexes.

## 2 Materials and Methods

### 2.1 *Drosophila* Lines and Culture

The *D. melanogaster* lines used in this study were derived from an experimental evolution model of aging and reproduction (Rose 1984; Carnes et al. 2015). Five replicate “O” lines were established through continued directional selection for late‐life female reproduction across hundreds of generations, resulting in significantly extended lifespan and delayed reproductive senescence. In parallel, five unselected “B” control lines derived from the same base population were maintained under standard laboratory conditions without age‐related reproductive selection and serve as a genetically matched baseline. All flies were reared on standard cornmeal–yeast–molasses medium at 25°C under a 12:12 h light–dark cycle.

For oxygen consumption rate assays, virgin flies were collected under light CO_2_ anesthesia and housed in same‐sex groups of 10–12 individuals per vial. Oxygen consumption was measured in young (3–5 days old), middle‐aged (18–20 days old), and old (5 weeks old) flies. Untreated flies were maintained on the same food as the treated groups, minus the drug. For the treatment groups, lisinopril was incorporated into the food at a concentration of 1 mM, and flies were exposed to the drug continuously from eclosion until the time of measurement.

### 2.2 Oxygen Consumption Rate Assay

Organismal oxygen consumption rate, used as a proxy for metabolic rate, was measured using a 24‐well plate respirometry system (Loligo Systems, Viborg, Denmark) at the University of Alabama at Birmingham Small Animal Phenotyping Core. A detailed protocol is provided in (Hagedorn et al. 2023). Briefly, oxygen levels from individual flies were recorded continuously at 1‐s intervals over a 60‐min period, with the final 30 min used for analysis following an initial 30‐min acclimation phase. Background respiration was subtracted using empty chambers run in parallel, and oxygen consumption rates were expressed as μmol O_2_ consumed per minute per milligram of body weight. All measurements were performed during the same circadian time window (ZT3–ZT5) to minimize variability due to diurnal fluctuations in metabolism. A total of 2213 individual flies were assessed for oxygen consumption rate, with 15–20 flies measured per group across five B lines and five O lines, two sexes, three age groups (young, middle‐aged, and old), and two treatment conditions (untreated and lisinopril‐treated).

To assess the effects of selection line, age, and sex on metabolic rate, we used a linear mixed‐effects model implemented in SAS 9.4 (SAS Institute Inc., Cary, NC, USA) using the PROC MIXED procedure. Fixed effects included selection line, age, sex, and all two‐ and three‐way interactions. For analyses that included pharmacological intervention, treatment (lisinopril) was also included as a fixed effect along with its interactions. Selection line replicate was included as a random effect to account for variability among independently evolved lines. *Post hoc* comparisons were performed using least‐squares means with Tukey adjustment for multiple testing. Statistical significance was set at *p* < 0.05.

### 2.3 Metabolomics Sample Processing

Metabolomic profiling of young and old male and female flies from the O and B lines was performed by Metabolon Inc. using their standardized ultra‐performance liquid chromatography–tandem mass spectrometry (UPLC‐MS/MS) platform. A total of 80 samples representing two replicates (each consisting of 100 pooled flies), two sexes, and five B and five O lines at two ages (3–5 days of age and 5 weeks of age) were collected in the Mackay laboratory. Samples were then shipped to Metabolon Inc. (North Carolina) for metabolomics analysis. Upon receipt, samples were accessioned into Metabolon's laboratory information management system (LIMS), assigned unique identifiers, and stored at −80°C until processing. Details are provided in Zhou et al. (2020). Briefly, sample preparation was performed using the automated MicroLab STAR system (Hamilton Company). Several internal standards were added to each sample for quality control (QC). Proteins were precipitated with methanol under vigorous shaking, and the resulting extracts were separated into four analytical fractions: two for reverse‐phase (RP) UPLC‐MS/MS with positive electrospray ionization (ESI), one for RP/UPLC‐MS/MS with negative ESI, and one for HILIC/UPLC‐MS/MS with negative ESI. Extracts were briefly evaporated under nitrogen and stored overnight prior to analysis. Quality assurance and control procedures included pooled matrix samples (technical replicates), process blanks (extracted water samples), and spiked internal standards to monitor retention time alignment and instrument performance. Instrument and process variability were assessed using the median relative standard deviation across instrument standards and endogenous compounds present in 100% of pooled matrix samples. Samples were randomized across the platform and ran with evenly spaced QC injections. Compound identification was based on three criteria: retention index, accurate mass (±10 ppm), and MS/MS spectral match scores (forward and reverse) compared to an in‐house library of over 3300 purified standards and recurrent unknowns. Matching consistency across samples was verified through proprietary software and manual review by Metabolon analysts. Data curation involved the removal of artifacts, misassignments, and background signals through proprietary software and manual verification. Peaks were quantified using the area under the curve. For multi‐day runs, block normalization was applied by adjusting compound medians per run‐day to 1.00 to correct inter‐day variability.

### 2.4 Metabolomics Statistical Analysis

Bradford protein concentrations and DNA concentrations were obtained for each sample. Protein concentrations varied little across the 80 samples, but DNA concentrations varied between males and females, even though an identical mass‐equivalent was extracted for each sample. Therefore, raw metabolite intensities were normalized to sample DNA concentration to reduce variation in the dataset as a whole, primarily in Principal Component 3, which accounts for 7%–10% of the variation (Figure S1). These data were log‐transformed prior to statistical analysis. Each metabolite was then rescaled by dividing by its median value across all samples, setting the median equal to 1 to facilitate cross‐sample comparisons. Missing values were imputed using the minimum observed value for each metabolite. Metabolites were grouped into super‐pathways and sub‐pathways based on Metabolon's internal biochemical classification system. Principal component analysis (PCA) and random forest analysis (RFA) were performed using Metabolon's proprietary bioinformatics platform to visualize global variation in metabolomic profiles and identify key discriminating metabolites. PCA was used to assess sample clustering by selection line, age, sex, and treatment. RFA ranked metabolites by their mean decrease in classification accuracy to identify those contributing most to group separation.

Metabolic pathway analysis was conducted using MetaboAnalyst 6.0 (available at https://www.metaboanalyst.ca/home.xhtml). The *D. melanogaster* KEGG metabolite set library was used as the background reference, and the Compound ID Conversion tool in the “Other Utilities” module of MetaboAnalyst was used to ensure compatibility of metabolite identifiers with the platform. Pathway enrichment was detected using hypergeometric testing with false discovery rate (FDR), and pathways with *q*‐values < 0.05 were considered significantly enriched.

A three‐way fixed effects analysis of variance (ANOVA) was used to assess the effects of selection line, age, and sex on individual metabolite levels, including their two‐ and three‐way interactions. FDR correction was applied to adjust for multiple comparisons, and metabolites with *q*‐values < 0.05 were considered statistically significant.

## 3 Results

### 3.1 Variation in Organismal Metabolic Rate Reveals Distinct Aging Trajectories in *D. melanogaster* O and B Lines

**FIGURE 1:**
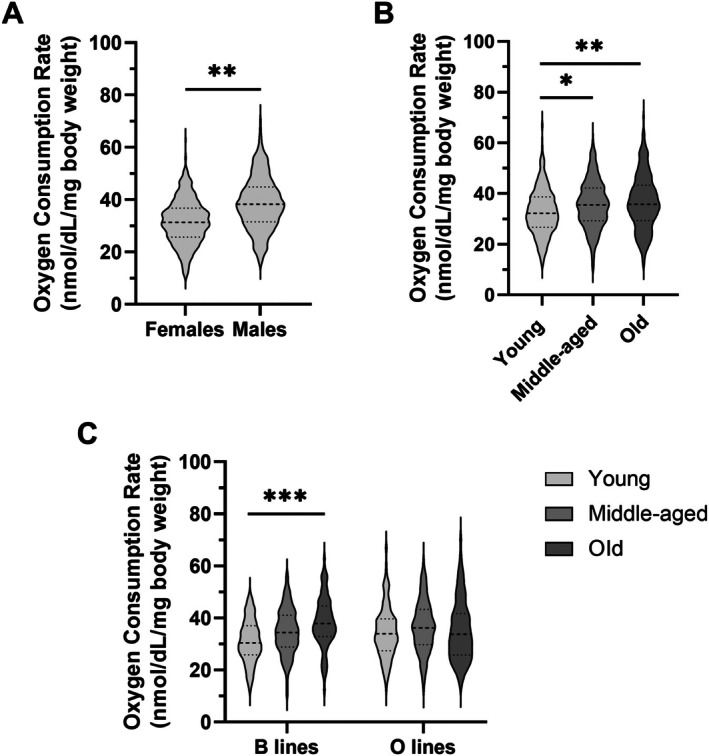
Organismal oxygen consumption rate increases with age in *D. melanogaster* B lines but remains stable in O lines. A three‐way ANOVA was used to test the effects of selection line, sex, and age, as well as their interactions, on oxygen consumption rate. Violin plots display the significant effects of sex (A), age (B), and line × age interaction (C). Plots show the distribution of oxygen consumption rate values, with the median indicated by a dashed line and the first and third quartiles by dotted lines. Asterisks indicate significance based on Tukey's post hoc tests: *p < 0.05, **p < 0.01, ***p < 0.001.

To monitor the organismal aging of *D. melanogaster* O and B lines, we measured metabolic rate at different time points (Table S1). A three‐way ANOVA revealed significant main effects of sex and age on organismal oxygen consumption (Table 1). Across all groups, males exhibited significantly higher (23%) metabolic rates than females (*p* = 0.0011; Figure 1A), consistent with a robust sex effect. We also observed a significant effect of age (*p* = 0.0014; Figure 1B), indicating that the metabolic rate varied across the lifespan. However, the trajectory of age‐related change differed by selection line. Although no significant main effect of line was detected, a significant line × age interaction (*p* = 0.0121; Table 1) indicates that aging influences metabolic rate differently in B and O lines. As shown in Figure 1C, oxygen consumption was significantly higher (24%) in old B line flies than in young flies (*p* = 0.0007), regardless of sex, while the O lines maintained stable metabolic rates across all age groups. These findings suggest that the overall age effect was primarily driven by increases in the B lines. The absence of age‐related metabolic increase in the O lines implies that selection for delayed reproduction may have buffered against the rise in metabolic rate observed during normal aging in *D. melanogaster* .

**TABLE 1:** Type 3 tests of fixed effects from ANOVA of organismal oxygen consumption in *Drosophila* O and B line females and males across young, middle‐aged, and old groups.

Effect	DF between groups	DF within groups	*F* value	*p*
Line	1	8	0.28	0.6140
Sex	1	8	25.00	**0.0011**
Age	2	16	10.11	**0.0014**
Line × sex	1	8	3.49	0.0988
Line × age	2	16	5.88	**0.0121**
Sex × age	2	16	0.10	0.9043
Line × sex × age	2	16	1.24	0.3146

*Note:* Bold font denotes statistical significance (*p* < 0.05).

Abbreviation: DF, degrees of freedom.

### 3.2 Aging‐Dependent Changes in One‐Carbon, Amino Acid, and Energy Metabolism Distinguish O and B Lines in Both Sexes

**FIGURE 2:**
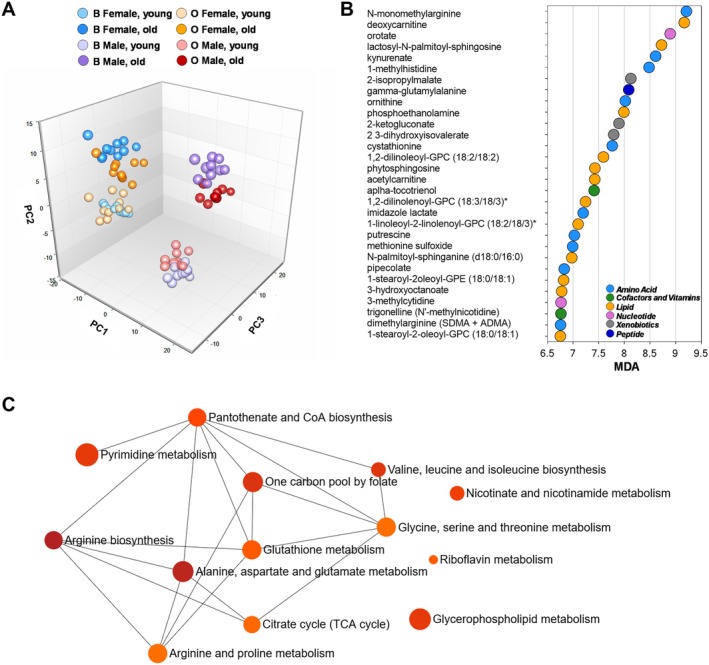
Metabolomic profiles reveal sex‐ and age‐dependent metabolic divergence between B and O lines, with pathways involving amino acid, methionine, one‐carbon metabolism, and the TCA cycle. (A) Three‐dimensional Principal Component Analysis (PCA) score plot of 451 metabolites from young and old male and female flies from the long‐lived O lines and the unselected B lines. Each point represents an individual sample, color‐coded by line, age, and sex. PC1 (39.6% of variance) separates samples primarily by sex. PC2 (11.4% of variance) captures age‐related differences, with broader dispersion among B line females along the *y*‐axis, indicating greater metabolic divergence with aging. In contrast, young and old O line females show relatively modest divergence. PC3 (7% of variance) also reflects age‐associated variation, with more pronounced separation among male flies along the *z*‐axis. (B) Supervised Random Forest Analysis was used to classify samples into eight predefined groups based on metabolite profiles: B line females (young and old), B line males (young and old), O line females (young and old), and O line males (young and old). The variable importance plot ranks metabolites by their contribution to classification accuracy, measured as Mean Decrease Accuracy (MDA). Metabolites with higher MDA values (toward the top of the plot) had the strongest influence on group discrimination. (C) Network visualization of enriched metabolic pathways identified through functional enrichment analysis using MetaboAnalyst 6.0. Each node represents a significantly enriched metabolite set (FDR‐adjusted *p* < 0.05); the node size reflects the number of matched metabolites (hits), and node color indicates statistical significance using a red‐to‐orange gradient, with dark red denoting the most significant enrichment and orange indicating less significant enrichment. Edges connect pathways that share common metabolites.

To explore the biochemical underpinnings of these physiological differences, we performed untargeted metabolomics profiling on young and old flies from both selection regimes. Following data preprocessing and filtering, a total of 451 metabolite features were detected (Table S2). Metabolite abundances were normalized to DNA content and classified into eight major “super pathways”, encompassing lipid metabolism, xenobiotic processing, nucleotide metabolism, amino acid metabolism, energy metabolism, carbohydrate metabolism, cofactors and vitamins, and peptide metabolism (Table S2). To assess global patterns in metabolite abundance, we first performed an unsupervised PCA on DNA‐normalized pooled metabolites. We found that PC1, which captures the greatest variance across the dataset (39.6%), clearly separated samples by sex, with male and female flies clustering distinctly across lines and ages (Figure 2A). PC2 (11.4%) separated samples according to age, particularly in the B line females and males, as well as in O males, where young and old flies were metabolically distinct. In contrast, O line females clustered tightly across age groups, consistent with metabolic stability across the lifespan. PC3, which accounted for an additional 7.0% of the total variance, also appeared to separate based on age, with a greater separation in males than in females, consistent with sex‐specific metabolic responses to aging (Figure 2A). To further validate group‐level metabolic distinctions and identify features driving group classification, we performed a supervised RFA using metabolite data from all eight experimental groups: B females (young and old), B males (young and old), O females (young and old), and O males (young and old). The model achieved a predictive accuracy of 99%, substantially exceeding the ~12% accuracy expected by random chance for an eight‐group classification. The top 30 discriminating metabolites included those involved in lipid metabolism (e.g., complex lipids and deoxycarnitine), the tryptophan pathway (e.g., kynurenate), arginine metabolism (e.g., N‐monomethylarginine, dimethylarginine, and putrescine), and redox homeostasis (e.g., gamma‐glutamylalanine and methionine sulfoxide) (Figure 2B). To uncover the fundamental metabolic processes shared across all biological groups, we subsequently ran pathway enrichment analysis. This revealed significant enrichment (FDR < 0.05) of pathways related to essential amino acid metabolism, including alanine, aspartate, and glutamate metabolism, arginine biosynthesis, and valine, leucine, and isoleucine biosynthesis (Table S3). These pathways formed a highly interconnected network with glycine, serine, and threonine metabolism, folate one‐carbon pool, glutathione metabolism, and tricarboxylic acid (TCA) cycle, revealing interdependence among shifts in amino acid turnover, methylation capacity, antioxidant defense, and mitochondrial function (Figure 2C). Three separate clusters of metabolites involved in glycerophospholipids, riboflavin, and nicotinate/nicotinamide metabolism were also identified (Figure 2C).

To identify age‐related metabolic signatures differing between B and O lines, we performed three‐way ANOVAs with selection history, age, and sex as main effects, along with all corresponding interaction effects. We identified 253, 438, and 321 metabolite features that differed significantly (FDR‐adjusted *p* < 0.05) between selection line, sex, and age, respectively (Table S4). Additionally, we detected 158 metabolites that exhibited differences between the B and O lines in an age‐specific manner, consistent with the observed line × age effect on whole‐organism oxygen consumption. This suggests a metabolic basis for the divergent aging trajectories of B and O lines across sexes. Among these 158 metabolites, 56 also varied by sex independent of genotype and age, and 38 displayed significant sex × age interaction effects independent of genotype. Notably, only 41 metabolites exhibited a line × sex × age interaction effect (Table S4), suggesting that the effect of aging on the metabolic profiles of B and O is largely similar in males and females.

**FIGURE 3:**
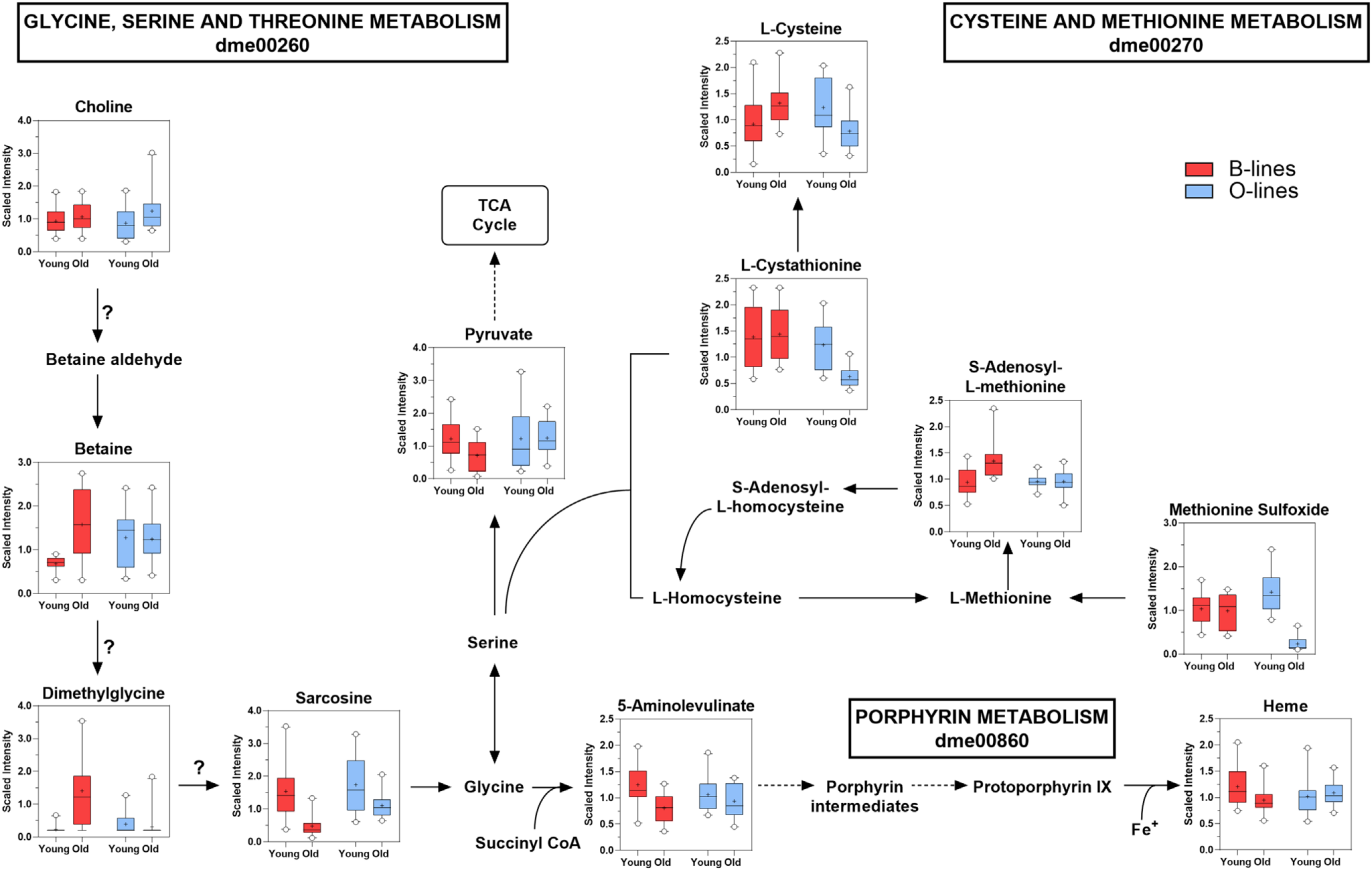
Distinct age‐related changes in methionine, one‐carbon, and porphyrin metabolism differentiate the B and O lines in both sexes. The schematic illustrates interconnected metabolic pathways involving glycine, serine, and threonine metabolism; cysteine and methionine metabolism; and porphyrin metabolism in *D. melanogaster* , based on KEGG architecture. All metabolites shown in the figure exhibited a significant line × age interaction effect, denoting genotype‐specific metabolic changes with aging in both sexes. Overlaid box‐and‐whisker plots display relative metabolite abundance across groups (young and old B and O lines). Arrows indicate known enzymatic reactions; dashed arrows represent multi‐step conversions; and question marks denote steps for which no corresponding enzyme has been annotated in *D. melanogaster* .

**FIGURE 4:**
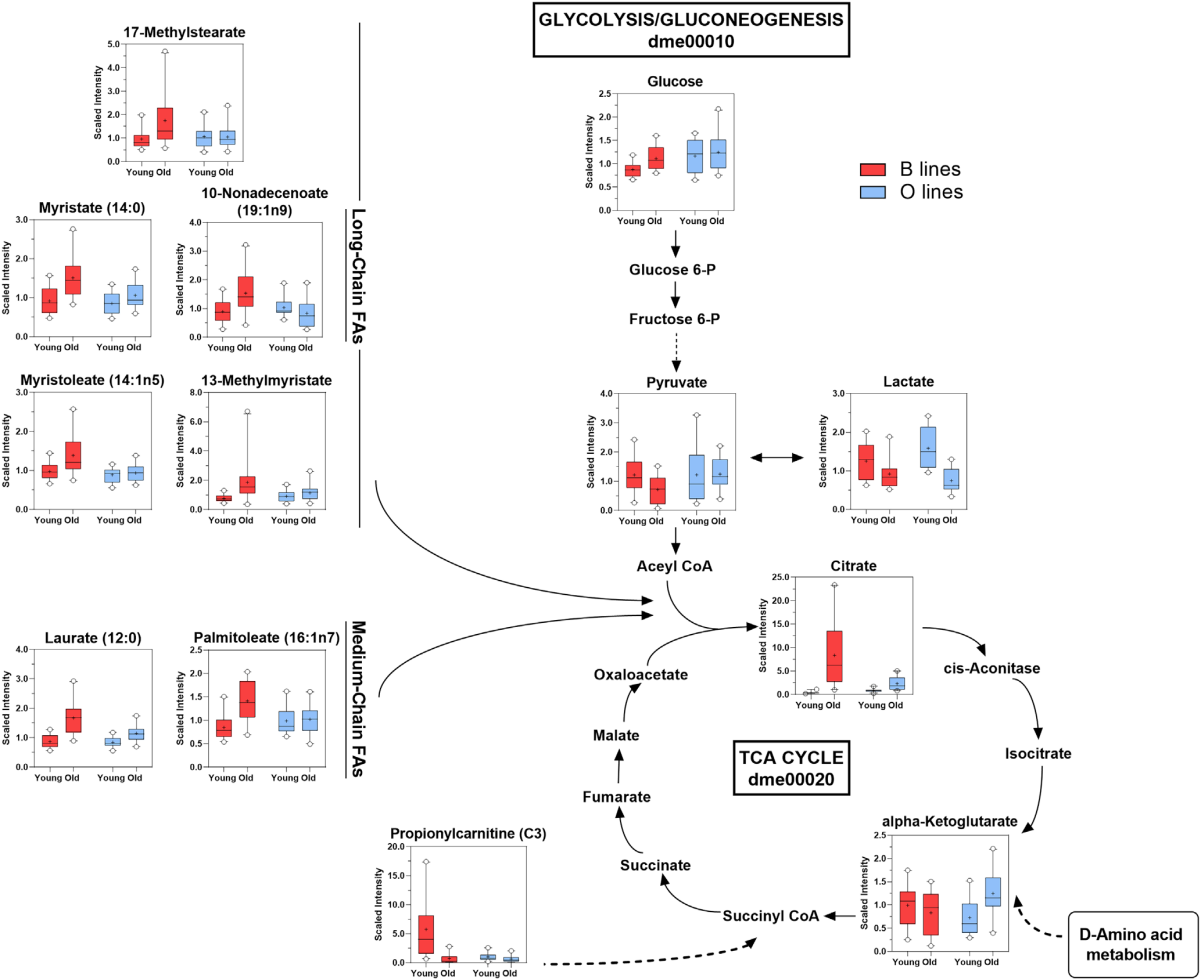
Age‐related accumulation of glucose, citrate, fatty acids, and propionylcarnitine in B lines reflects divergent remodeling of central carbon and lipid metabolism in both sexes. The schematic illustrates central carbon metabolism, including glycolysis/gluconeogenesis, the tricarboxylic acid (TCA) cycle (based on KEGG architecture), and associated lipid pathways in *D. melanogaster* . Metabolites included in the figure showed a significant line × age interaction effect, denoting genotype‐specific metabolic changes with aging in both sexes. Overlaid box‐and‐whisker plots display relative metabolite abundance across groups (young and old B and O lines). Aged B lines exhibited elevated levels of glucose, citrate, long‐chain fatty acids, and propionylcarnitine, suggesting altered carbon flux, impaired mitochondrial substrate utilization, and lipid accumulation, while O lines maintained a more stable metabolic profile. Arrows indicate known enzymatic reactions, and dashed arrows represent multi‐step conversions.

Consistent with the pathway‐level distinctions, aged B flies exhibited elevated levels of betaine, dimethylglycine, and S‐adenosylmethionine (SAM), alongside reductions in sarcosine and 5‐aminolevulinate compared to their younger counterparts (Figure 3). These metabolite changes suggest altered one‐carbon metabolism and point to a potential bottleneck in the dimethylglycine‐to‐sarcosine conversion step. However, this interpretation should be made cautiously, as the gene encoding the relevant enzyme has not yet been identified in *Drosophila*. The decline in 5‐aminolevulinate, a key precursor in heme biosynthesis, was accompanied by reduced heme levels, further supporting impaired heme production in aged B flies. Additionally, aged B flies showed increased glucose and citrate, modest reductions in pyruvate and lactate, and lower levels of propionylcarnitine (C3), a short‐chain acylcarnitine involved in mitochondrial import of propionyl groups (Siliprandi et al. 1991) (Figure 4). Together, these features suggest diminished glycolytic flux and disrupted mitochondrial function in the aged B flies. Elevated levels of several medium‐chain and monounsaturated fatty acids, including 17‐methylstearate, myristate, 10‐nonadecenoate (19:1n9), 13‐methylmyristate, laurate (12:0), and palmitoleate (16:1n7), further point to altered lipid metabolism and possible impairments in β‐oxidation with normal aging (Figure 4).

In contrast, aged O flies displayed a modest increase in choline and reductions in cysteine, cystathionine, and methionine sulfoxide, suggesting changes in transsulfuration and improved redox homeostasis (Figure 3). This pattern may reflect a reduced reliance on transsulfuration for maintaining redox balance, consistent with lower oxidative burden and enhanced metabolic stability. Moreover, lactate levels were decreased, and alpha‐ketoglutarate levels were elevated, indicative of sustained TCA cycle activity and preserved metabolic flexibility through anaplerotic input (Figure 4). Together, these findings support the hypothesis that O lines maintain more resilient energy metabolism during aging, consistent with their stable organismal oxygen consumption trajectories.

**FIGURE 5:**
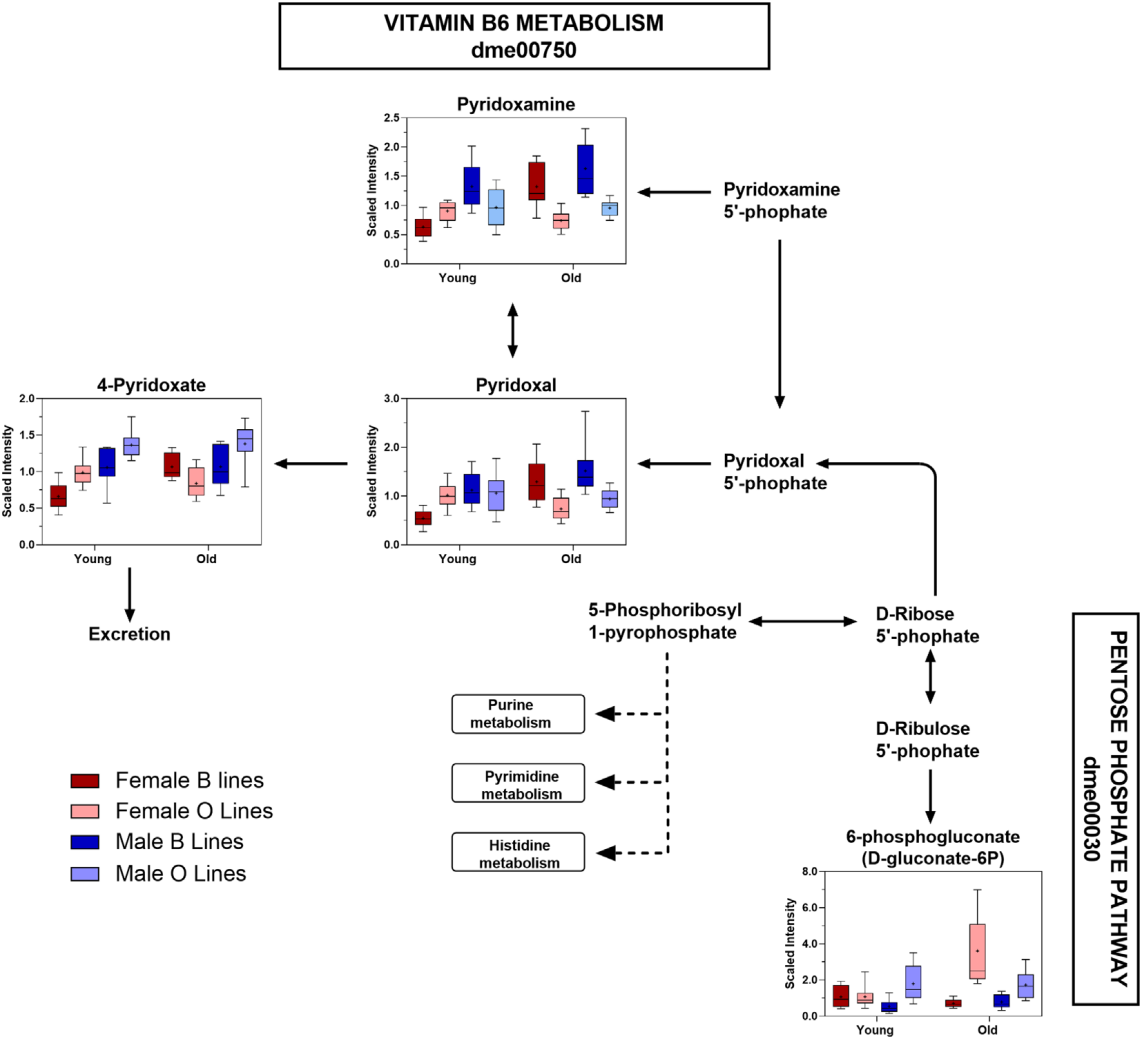
Vitamin B6 metabolism is differentially altered with age and genotype and displays distinct effects in females. The schematic illustrates Vitamin B6 metabolism and its link to the pentose phosphate pathway (based on KEGG architecture) in *D. melanogaster* . All metabolites shown in the figure had a significant line × sex × age interaction effect. Boxplots display scaled intensities for each metabolite grouped by young and old individuals in B females, O females, B males, and O males. In B females, pyridoxamine, pyridoxal, and 4‐pyridoxate increase with age, but these metabolites remain unchanged in O females. In contrast, 6‐phosphogluconate is stable in B females but increases with aging in O females. Arrows indicate known enzymatic reactions, and dashed arrows represent multi‐step conversions.

Among the 41 metabolites that differed between B and O lines in a sex‐ and age‐specific manner, we found that B and O females showed distinct metabolic responses to aging within the Vitamin B6 pathway. In B females, pyridoxamine, pyridoxal, and 4‐pyridoxate increased with age, whereas these metabolites remained unchanged in O females (Figure 5). Additionally, aged O females showed an accumulation of 6‐phosphogluconate, while levels in B females remained stable. These patterns indicate genotype‐specific differences in age‐related changes across both Vitamin B6 metabolism and the pentose phosphate pathway.

### 3.3 Lisinopril Modulates Age‐Related Metabolic Rate Trajectories in a Sex‐Specific Manner

**FIGURE 6:**
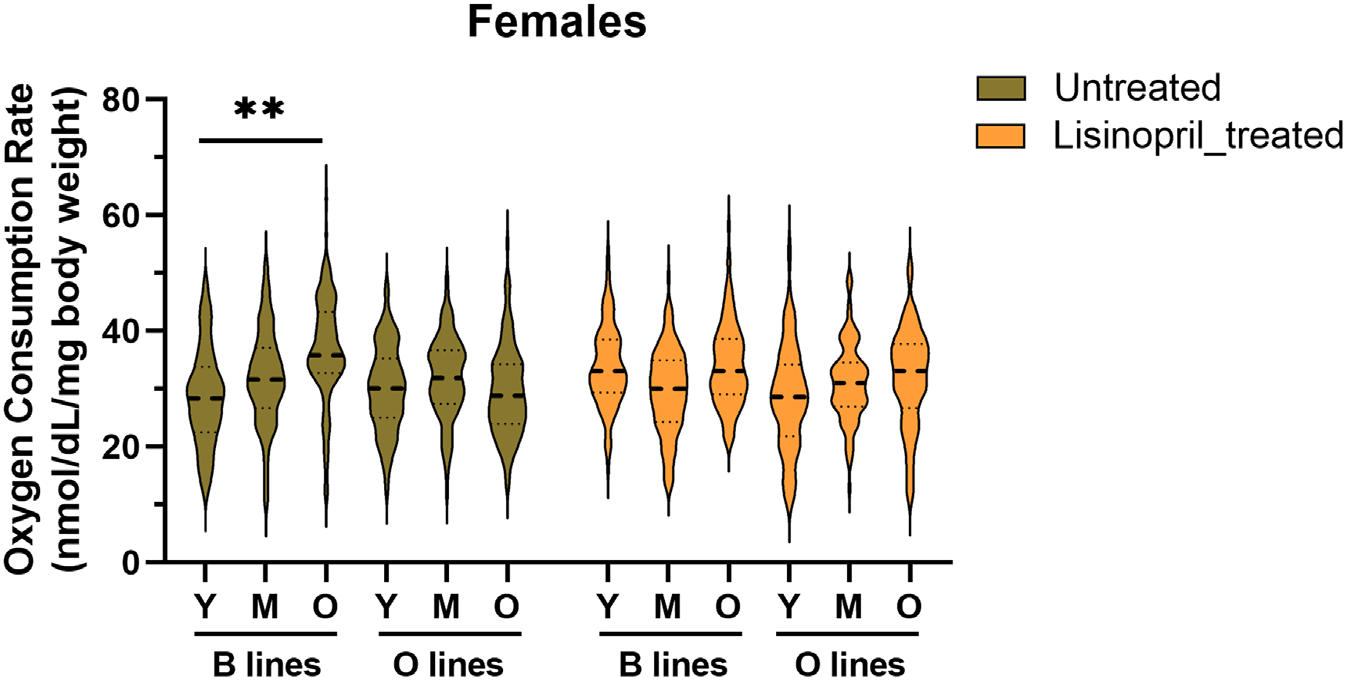
Lisinopril treatment blunts the age‐related increase in metabolic rate in B line females. The graph illustrates the significant line × treatment × age interaction identified in females from a three‐way ANOVA, performed on data stratified by sex. Violin plots depict the distribution of oxygen consumption rate values across young (Y), middle‐aged (M), and old (O) female flies from B and O lines, either untreated or treated with Lisinopril, with the median indicated by a dashed line and the first and third quartiles by dotted lines. Old untreated B line females showed a significant increase in oxygen consumption compared to young B females. In contrast, Lisinopril‐treated B line females maintained stable metabolic rates with age. The oxygen consumption rate in O line females remained relatively stable across age and treatment groups. ** indicates *p* < 0.01, based on Tukey's post hoc test.

To evaluate the effects of ACE inhibition on organismal oxygen consumption rate, we treated male and female flies from both young, middle‐aged, and old cohorts of the B and O lines with lisinopril. Given prior evidence of sex‐specific responses to ACE inhibition (Vecchie et al. 2024), we analyzed oxygen consumption data stratified by sex to account for potential differences. In females, there was no effect of treatment (Table S5); however, we found a significant line × treatment × age interaction effect (*p* = 0.0069), indicating that the metabolic response to aging and lisinopril varied by line. In contrast to the clear age‐associated increase in metabolic rate observed in untreated B line females, lisinopril‐treated B line females did not exhibit this increase, instead maintaining a relatively stable metabolic rate across life stages (Figure 6). This pattern resembled both untreated and treated O line females, whose metabolic rates remained stable with age regardless of treatment (Figure 6). These results suggest that lisinopril blunts the age‐related increase in metabolic rate observed in B line females.

Contrary to females, we detected a significant main effect of treatment (*p* = 0.0173), with no significant interactions in males (Table S5). Lisinopril treatment increased metabolic rate in males, regardless of line or life stage (Untreated: 38.67 ± 0.89 nmL/mg body weight; lisinopril‐treated: 41.60 ± 0.90 nmL/mg body weight; *p* = 0.0173). This finding contrasts with the buffering effect observed in females and suggests that ACE inhibition enhances metabolic rate in males regardless of age and genotype, pointing to a sex‐dependent physiological response to lisinopril.

## 4 Discussion

Aging is accompanied by complex physiological and metabolic changes, yet the trajectory of these changes can be influenced by genetic background and evolutionary history (Giuliani et al. 2018). The long‐lived O lines and their B line controls provide a unique opportunity to investigate how selection for delayed reproduction (and the corresponding correlated response for increased lifespan) shapes metabolic resilience in the genetically tractable *Drosophila* model. Here, we demonstrate that aging leads to a significant increase in metabolic rate in the B lines, but not in the increased longevity‐selected O lines. This divergence highlights fundamental differences in how these genotypes regulate energy metabolism across the lifespan. In the B lines, which evolved under selection for early‐life reproduction, the age‐related increase in metabolic rate may reflect a compensatory response to declining mitochondrial efficiency (Celotto et al. 2011). Increased oxygen consumption may also indicate a loss of mitochondrial coupling efficiency, whereby more oxygen is consumed per unit of ATP produced, leading to a state of bioenergetic inefficiency (Demine et al. 2019). Sustained high metabolic demands may further deplete internal energy reserves, such as lipids, glycogen, and amino acids, thereby compromising the organism's ability to maintain homeostasis or respond to physiological stress in late life (Moldakozhayev and Gladyshev 2023). Notably, our findings in the B flies are consistent with human genetic evidence showing that individuals with a higher genetically predicted basal metabolic rate tend to have parents with shorter lifespans (Ng and Schooling 2023).

To further characterize the physiological differences and investigate the metabolic basis of divergent aging trajectories in the O and B lines, we performed untargeted metabolomics in young and old male and female flies. PCA revealed sex as the dominant source of variation, while age‐related metabolic differences were most pronounced in B lines and O males. In contrast, O females clustered tightly across age groups, consistent with preserved metabolic stability. RFA confirmed robust group separation and identified key discriminating metabolites related to lipid metabolism, methylated arginines, and redox regulation. These results reveal both conserved and genotype‐specific metabolic features of aging and point to candidate biomarkers and pathways that may underline the physiological divergence between O and B lines. In this regard, our pathway‐level analysis revealed a tightly interconnected metabolic network involving glycine–serine–threonine metabolism, one‐carbon metabolism, and the TCA cycle. This interdependence implies that innate and environmental changes arising with age (or selection) act on shared metabolic nodes, producing system‐wide adjustments rather than pathway‐specific effects. The relevance of this core metabolic framework for lifespan appears to be evolutionarily conserved, as the one‐carbon pathways linked to glycine, serine, and threonine metabolism have also been identified as key pathways influencing lifespan in mice (Aon et al. 2020).

In our study, we also detected distinct age‐related metabolic signatures in the B and O lines. We found that aged B lines had elevated levels of SAM, betaine, and dimethylglycine, along with reduced levels of sarcosine in both sexes, suggesting disrupted one‐carbon metabolism and potential methylation overload (Obeid 2013). Aged B flies also showed reduced levels of 5‐aminolevulinate and heme, indicating impaired heme biosynthesis (Hunter and Ferreira 2011). Given that heme is involved in mitochondrial electron transport and redox regulation (Yien and Perfetto 2022), its depletion may contribute to diminished oxidative capacity and elevated oxidative stress. Enhancing SAM catabolism via glycine‐N‐methyltransferase overexpression or suppression of S‐adenosyl‐L‐homocysteine hydrolase extends longevity in *Drosophila* (Obata and Miura 2015; Parkhitko et al. 2016). Similarly, dietary methionine restriction, which lowers SAM levels, promotes metabolic health and lifespan extension across species (Kitada et al. 2021). Recent work has also demonstrated that promoting heme synthesis or supplementation extends lifespan in yeast (Patnaik et al. 2024). Our results corroborate the growing view that disruption of one‐carbon and heme metabolism contributes to aging.

The alterations in one‐carbon and heme metabolism were accompanied by elevated glucose and citrate levels, reduced pyruvate and lactate, and accumulation of medium‐ and long‐chain fatty acids. The buildup of these fatty acids likely reflects impaired mitochondrial fatty acid oxidation rather than increased substrate availability or utilization. This interpretation is supported by decreased levels of propionylcarnitine (C3), a product of odd‐chain fatty acid and branched‐chain amino acid catabolism (Siliprandi et al. 1991), pointing to reduced mitochondrial β‐oxidation and limited anaplerotic input into the TCA cycle (Lerin et al. 2016). Elevated citrate may indicate a bottleneck in TCA cycle flux or impaired aconitase activity, a redox‐sensitive enzyme known to decline with age (Yan et al. 1997), and may further inhibit glycolysis through feedback inhibition of phosphofructokinase. Our findings align with previous work demonstrating that *Drosophila* metabolism is highly responsive to internal physiological states. Wilinski et al. (2019) reported that rapid and tissue‐specific metabolic shifts occur during transitions from hunger to satiety, and that high‐sugar diets dampen both behavioral and metabolic responses to feeding. Notably, suppression of one‐carbon metabolism and remodeling of the TCA cycle emerged as key features of diet‐induced impairments in the ability to switch between fuel sources. While their study focused on acute dietary effects, our findings extend this concept to the genotype‐driven metabolic rigidity that emerges with age in the B lines. The metabolic profile of aged B flies, marked by disrupted one‐carbon and mitochondrial metabolism, suggests a diminished capacity to adaptively coordinate nutrient flux and meet changing energetic demands.

The metabolomic signatures observed in aged B lines contrast sharply with those of the O lines, which maintained stable levels of glycolytic and TCA cycle intermediates, elevated alpha‐ketoglutarate, and reduced lactate, features indicative of preserved oxidative metabolism and reduced reliance on anaerobic glycolysis. Aged O flies also exhibited lower levels of transsulfuration intermediates and oxidative stress markers, including cysteine, cystathionine, and methionine sulfoxide, consistent with enhanced redox efficiency and a reduced oxidative burden. We previously showed that aged O lines consume more food than B lines (Carnes et al. 2015), suggesting that their elevated metabolic capacity is matched by increased energy intake. Rather than reflecting a downregulation of energy use, the longevity phenotype of the O lines appears to involve a coordinated enhancement of energy acquisition, efficiency, and metabolic homeostasis. This strategy likely enables extended reproductive capacity and delayed senescence, consistent with the disposable soma theory (Kirkwood and Holliday 1979), which posits that energy is differentially allocated between reproduction and somatic maintenance to maximize fitness over the lifespan. This idea is further supported by the distinct metabolic responses to aging observed between B and O females in Vitamin B6 metabolites. B females showed age‐related increases in pyridoxamine, pyridoxal, and 4‐pyridoxate, consistent with activation of B6‐dependent pathways that support reproductive maturation (Yu et al. 2023). In contrast, these metabolites remained unchanged with aging in O females, suggesting they transition more slowly into a reproductive metabolic state. At the same time, aged O females accumulate 6‐phosphogluconate, which may indicate reduced pentose phosphate pathway activity and limited NADPH production. Such a metabolic bottleneck would further restrict the shift toward anabolic processes required for reproduction. Although correlational, these patterns are consistent with O females prioritizing somatic maintenance over early reproductive effort, contributing to their delayed reproductive timing.

Our findings extend previous work showing that the evolution of longevity is associated with preserved mitochondrial function and resistance to age‐related metabolic drift (Hubert et al. 2025; Phillips et al. 2022). The convergence of results across independently evolved lines supports the broader conclusion that selection on reproductive timing drives predictable remodeling of core metabolic networks. However, while prior studies established genetic and biochemical correlates of longevity, the physiological consequences remained less well defined. By integrating whole‐organism metabolic rate measurements with untargeted metabolomics, our study provides a systems‐level view of how delayed reproduction reshapes energy metabolism to support healthy aging.

In an earlier study, we reported that lisinopril, an ACE inhibitor, modulates key features of metabolic aging in the thoraces of *D. melanogaster* in a genotype‐ and age‐dependent manner (Ederer et al. 2018). Lisinopril reduces mitochondrial oxygen consumption in young flies, increases mitochondrial content in middle‐aged flies, and decreases reactive oxygen species levels, suggesting an influence on mitochondrial efficiency and redox balance. We also linked lisinopril to changes in glycolysis, glycogen degradation, and mevalonate metabolism, pathways central to energy and membrane homeostasis (Ederer et al. 2018). In the present study, we extend these findings by showing that lisinopril attenuates the age‐associated increase in metabolic rate observed in B line females, effectively phenocopying the metabolically stable profile of O line females. This suggests that ACE inhibition may help preserve metabolic function during aging, particularly in genotypes lacking intrinsic protection against metabolic decline.

In contrast to females, lisinopril treatment significantly increased metabolic rate in males across both genotypes and life stages, indicating a robust and antagonistic physiological effect. One possible explanation for these sex‐specific effects is that only females in the O lines were subject to selection for delayed reproduction, whereas males were not directly selected. Although males can exhibit correlated responses to female‐limited selection, the absence of direct evolutionary pressure may leave them more susceptible to pharmacological modulation of metabolic pathways.

A limitation of the study is that we did not examine the effect of lisinopril on lifespan in B and O flies, although we have previously shown that lisinopril extends lifespan in *D. melanogaster* (Gabrawy et al. 2019). Another limitation is that we did not test whether the metabolic changes observed here translate into differences in lifespan. Determining whether these metabolic changes contribute to longevity in B and O flies will be an important goal for future work. Additional studies are also needed to clarify the mechanisms through which lisinopril influences aging‐related metabolic changes. These should include direct assessments of mitochondrial function across age and genotype, genetic interaction experiments targeting evolutionarily conserved components of the RAS pathway, and multi‐omic integration (e.g., transcriptomics, proteomics) to uncover regulatory pathways modulated by lisinopril.

## 5 Conclusion

Our findings reveal that selection for delayed reproduction in females not only stabilizes metabolic rate but also reshapes the metabolome of both sexes in a manner consistent with energy efficiency and aging resistance. Our data suggest that pharmacological interventions, such as lisinopril, can mimic or modify metabolic rate in a sex‐specific fashion, raising the possibility that RAS‐targeting strategies could be leveraged to support metabolic health during aging. Further, our results support the broader concept that metabolic health during aging is not merely a function of reduced energy expenditure, but of strategically evolved energy management that sustains function and buffers against the physiological costs of aging.

## Author Contributions

Maria De Luca, Trudy F.C. Mackay, and Robert R.H. Anholt conceptualized and designed the study. Denise Vecchié performed the experiments. Maria De Luca and Trudy F.C. Mackay analyzed and interpreted the data. Maria De Luca drafted the manuscript. All authors reviewed and approved the final version of the manuscript.

## Funding

This work was supported by the National Institute on Aging grant R01 AG073181 awarded to Trudy F.C. Mackay, Maria De Luca, and Robert R.H. Anholt, R01 grant AG043490 awarded to Trudy F.C. Mackay and Robert R.H. Anholt, and the UAB Nathan Shock Center P30 grant AG050886.

## Conflicts of Interest

The authors declare no conflicts of interest.

## Supporting information


**Figure S1:** Principal components analysis of normalization methods for all metabolite data. Light blue: 3–5‐day old females, B lines. Dark blue: 5‐week‐old females, B lines. Light purple: 3–5‐day old males, B lines. Dark purple: 5‐week‐old males, B lines. Light orange: 3–5‐day old females, O lines. Dark orange: 5‐week‐old females, O lines. Light red: 3–5‐day old males, O lines. Dark red: 5‐week‐old males, O lines. (A) Non‐normalized. (B) Normalized to protein content. (C) Normalized to DNA content.

**Table S1:** Organismal oxygen consumption rate (OCR) in five selection replicates of female and male B and O flies measured at 1 week (young), 3 weeks (middle‐aged), and 5 weeks (old) of age under lisinopril treatment or untreated conditions.

**Table S2:** For each sample, metabolite values were normalized to DNA concentration and then log‐transformed for statistical analyses. [Biochemical Name]* indicates compounds that have not been officially confirmed based on a standard, but we are confident in its identity. Each metabolite is annotated with a super pathway and sub pathway, based on Metabolon's proprietary classification system.

**Table S3:** Metabolic pathway analysis performed with MetaboAnalyst using the enrichment hypergeometric test and the *D. melanogaster* KEGG pathway library. Total refers to the number of metabolites in each metabolite set. Expected indicates the number of overlapping metabolites expected by chance. Hits represents the number of input metabolites overlapping with the metabolite set. Significantly enriched sets were identified using a false discovery rate (FDR)‐adjusted *p*‐value threshold of < 0.05.

**Table S4:** A three‐way ANOVA of DNA‐normalized and log‐transformed metabolites was performed to assess the main effects of selection line, sex, and age, as well as their interactions. The table reports the raw *p*‐values and corresponding *q*‐values (FDR‐adjusted *p*‐values) for each factor. Each metabolite is annotated with a Super pathway and Sub pathway, based on Metabolon's proprietary classification system. B denotes the five B lines and O denotes the five O lines. W1 and W5 denotes weeks one and five, respectively. F indicates females and M males.

**Table S5:** Type 3 tests of fixed effects from ANOVA of organismal oxygen consumption in *Drosophila* O and B line females (A) and males (B) treated with lisinopril or untreated across age groups.

## Data Availability

The organismal oxygen consumption and metabolomic data supporting the findings of this study are available in the Supporting Information of this article.
