# Supplementary material for: Selection for Postponed Senescence in Drosophila melanogaster Reveals Distinct Metabolic Aging Trajectories Modifiable by the Angiotensin‐Converting Enzyme Inhibitor Lisinopril
Source: Aging Cell. 2026 Jan 14;25(2):e70375. doi: 10.1111/acel.70375 (PMC12803505; doi:10.1111/acel.70375)
Supplement: Supplementary file 1 — Figure S1: Principal components analysis of normalization methods for all metabolite data. Light blue: 3–5‐day old females, B lines. Dark blue: 5‐week‐old females, B lines. Light purple: 3–5‐day old males, B lines. Dark purple: 5‐week‐old males, B lines. Light orange: 3–5‐day old females, O lines. Dark orange: 5‐week‐old females, O lines. Light red: 3–5‐day old males, O lines. Dark red: 5‐week‐old males, O lines. (A) Non‐normalized. (B) Normalized to protein content. (C) Normalized to DNA content. [file ACEL-25-e70375-s006.docx]

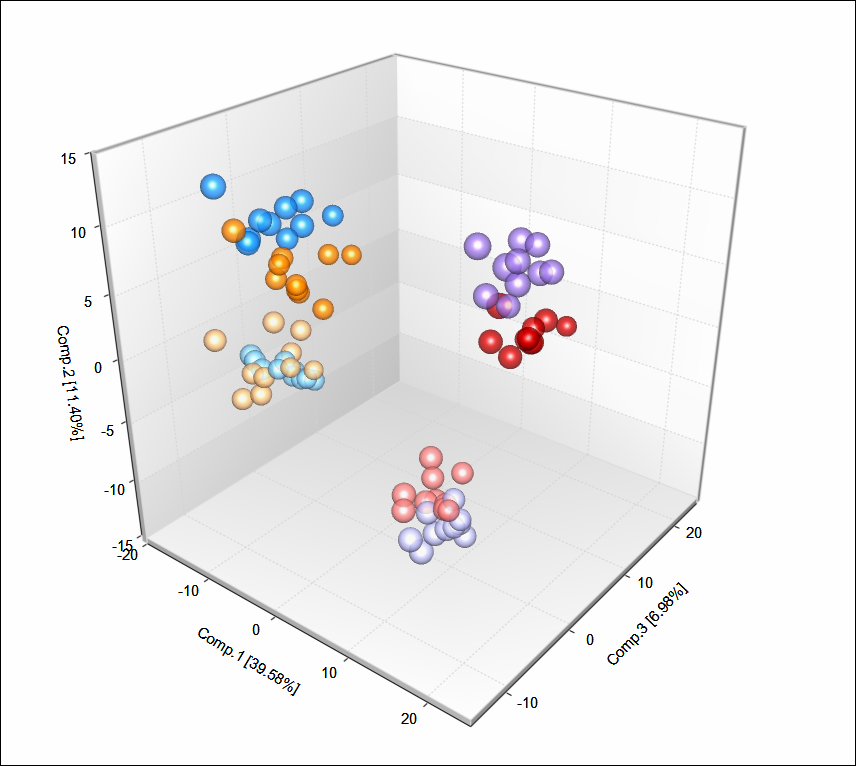


**A**

**B**

**C**

**Figure S1. Principal components analysis of normalization methods for all metabolite data.** Light blue: 3–5-day old females, B lines. Dark blue: 5-week-old females, B lines. Light purple: 3–5-day old males, B lines. Dark purple: 5-week-old males, B lines. Light orange: 3–5-day old females, O lines. Dark orange: 5-week-old females, O lines. Light red: 3–5-day old males, O lines. Dark red: 5-week-old males, O lines. (A) Non-normalized. (B) Normalized to protein content. (C) Normalized to DNA content.
