# Supplementary material for: Selection for Postponed Senescence in Drosophila melanogaster Reveals Distinct Metabolic Aging Trajectories Modifiable by the Angiotensin‐Converting Enzyme Inhibitor Lisinopril
Source: Aging Cell. 2026 Jan 14;25(2):e70375. doi: 10.1111/acel.70375 (PMC12803505; doi:10.1111/acel.70375)
Supplement: Supplementary file 6 — Table S5: Type 3 tests of fixed effects from ANOVA of organismal oxygen consumption in Drosophila O and B line females (A) and males (B) treated with lisinopril or untreated across age groups. [file ACEL-25-e70375-s001.docx]

**Supplementary Table S5**. Type 3 tests of fixed effects from ANOVA of organismal oxygen consumption in *Drosophila* O and B line females (A) and males (B) treated with lisinopril or untreated across age groups.

| **Effect** | **DF between groups** | **DF within groups** | **F value** | ***p*-value** |
| --- | --- | --- | --- | --- |
| **A** |  |  |  |  |
| Line | 1 | 8 | 2.06 | 0.1893 |
| Treat | 1 | 8 | 0.01 | 0.9113 |
| Age | 2 | 16 | 5.13 | **0.0190** |
| Line*Treat | 1 | 8 | 0.01 | 0.9368 |
| Line*Age | 2 | 16 | 4.99 | **0.0207** |
| Treat*Age | 2 | 16 | 2.21 | 0.1424 |
| Line*Treat*Age | 2 | 16 | 6.90 | **0.0069** |
| **B** |  |  |  |  |
| Line | 1 | 8 | 4.72 | 0.0617 |
| Treat | 1 | 8 | 8.94 | **0.0173** |
| Age | 2 | 16 | 9.61 | **0.0018** |
| Line*Treat | 1 | 8 | 0.12 | 0.7429 |
| Line*Age | 2 | 16 | 1.23 | 0.3175 |
| Treat*Age | 2 | 16 | 0.07 | 0.9281 |
| Line*Treat*Age | 2 | 16 | 0.10 | 0.9041 |

Abbreviation: DF: Degrees of freedom. Bold font denotes statistical significance (*p* < 0.05).
